# Bacteriology

**Published:** 1887-09

**Authors:** 


### Bacteriology.

W. Watson Cheyne, M. B., F. R. C. S., of London, concludes his third paper, on the relation of bacteria to the living animal body, in the *American Journal of Medical Science*, as follows:

"The local effects of bacteria are very various, and differ in the case of almost every bacterium. They may also, in most cases, be explained by supposing a local action of a poison originating as the result of the growth of the bacteria. The poison, however, is evidently not the same in all cases—indeed it is probably different in every instance. The most violent of the local effects of bacteria are seen in various gangrenous affections (acute traumatic gangrene and noma). Here it is not a case of death of the tissues as the result of violent inflammation, so much as a direct killing of them by the products of the bacteria. In acute spreading gangrene bacilli have been found which are apparently the cause of the disease. In noma long bacilli are present, which Mr. Lingard has demonstrated to be the cause of the disease. In gangrenous stomatitis in the calf, which affects calves at particular seasons, he has found bacilli which are very similar in appearance to those present in noma in man. On cultivation they present characters which render them easily distinguishable from other bacteria, and on inoculation of these organisms into the calf a gangrenous stomatitis is again produced.

A somewhat similar affection, though less violent, was studied by Koch in connection with mice, in which a progressive gangrene of the tissues was caused by the growth of a streptococcus in them.

Another set of local phenomena caused by bacteria are inflammatory, and in connection with these micrococci of various kinds are generally present. In inflammation in man two kinds of micrococci are chiefly found: streptococci, where the individual cocci are arranged in chains, and staphylococci, where they are arranged in groups. Of the latter there are several kinds. The streptococci are generally found in connection with violent phlegmonous inflammation, where there is a tendency to necrosis of tissue with but little suppuration; the staphylococci are chiefly found in abscesses. The conditions under which the abscesses arise in connection with these organisms are not very clear. I believe that local injury or weakening of the part is necessary in most cases, and that the primary dose is of great importance. That these organisms act by the production of poisonous or irritating materials seems certain, for if injected into the blood-stream in animals (and in man in pyæmia and ulcerative endocarditis) they give rise to emboli in various organs, and in stained preparations of these organs it is seen that the tissues in the immediate vicinity of the embolus do not stain, they are dead (coagulation-necrosis), while at some distance off a ring of leucocytes is formed which encloses the organisms. It is clear that the tissue in the immediate vicinity of the mass has been killed by some poisonous excretion, which, at a further distance, has been more dilute, and has acted only as an irritant. In connection with the pyogenic micrococci, it is interesting to observe that the same organisms are found in pyæmia, more especially the streptococci. Probably the difference in dose plays an important part in the different action of these micrococci, nevertheless I can not but think that some other conditions, with which we are as yet unacquainted, must come into play.

A third set of phenomena may be observed as the result of the local action of bacteria, viz.: formative and degenerative processes. In tubercle we have something more than merely inflammatory process; we have a distinct formation and transformation of cells as indicated by the appearance of large numbers of epithelial cells, and also of giant cells at an early age of the process. The tubercle bacillus seems at first to grow by preference (where it can) in epithelial (as in the alveoli of the lung) or endothelial cells, leading, in the first instance to their hypertrophy and multiplication, but, bye-and-bye, to their degeneration and caseous transformation. In the case of some cells, however, the vitality of the cell seems to be unusually great, and thus they go on growing, leading to the formation of a giant cell. A tubercle illustrates very well the fight between cells and bacilli. If the cells are the weaker they quickly die and undergo degeneration, and thus the center of the tubercle becomes converted into a caseous mass, in which the bacilli may for a short time continue to grow. If, however, the cell obtains the victory, the bacilli soon disappear, and a " fibrous tubercle," containing giant cells is left, in which few or no bacilli can be found. These are the tubercles which, on account of the absence or small number of the bacilli, have led to the formation of various theories as to tubercle being due to chemical poison, etc. These theories, however, depend, I believe on misinterpretation of the facts; such tubercles are rather to be regarded as tubercles in which the bacilli have been already destroyed than as growing nodules. Other examples of the formative action of bacteria are also seen in syphilitic affections and in leprosy."

